# Online searches for hepatocellular carcinoma drugs mirror prescription trends across specialties and changes in guideline recommendations

**DOI:** 10.3389/fonc.2024.1324095

**Published:** 2024-02-09

**Authors:** Philipp Berning, Adrian E. Schroer, Rishav Adhikari, Alexander C. Razavi, Francois H. Cornelis, Joseph P. Erinjeri, Stephen B. Solomon, Debkumar Sarkar, Hebert Alberto Vargas, Heiko Schöder, Josef J. Fox, Omar Dzaye

**Affiliations:** ^1^ Johns Hopkins Ciccarone Center for the Prevention of Cardiovascular Disease, Johns Hopkins University School of Medicine, Baltimore, MD, United States; ^2^ Department of Radiology, Memorial Sloan Kettering Cancer Center, New York, NY, United States; ^3^ Department of Radiology, NYU Langone Health, New York, NY, United States

**Keywords:** hepatocellular carcinoma, google trends, sorafenib, lenvatinib, cabozantinib

## Abstract

**Background & aims:**

The treatment options for systemically progressed hepatocellular carcinoma (HCC) have significantly expanded in recent years. In this study, we aimed to evaluate the potential of Google searches as a reflection of prescription rates for HCC drugs in the United States (US).

**Methods:**

We conducted an in-depth analysis of US prescription data obtained from the IQVIA National Prescription Audit (NPA) and corresponding Google Trends data from January 2017 to December 2022. We focused on drugs used in the first line and second or later treatment lines for HCC, collecting data on their prescriptions and search rates. Search volumes were collected as aggregated search queries for both generic drugs and their respective brand names.

**Results:**

During the study period from Q1 2017 to Q4 2022, monthly prescriptions for drugs used in HCC treatment showed an 173% increase (from 1253 to 3422). Conversely online searches increased by 3.5% (from 173 to 179 per 10 million searches). Notably, strong correlations were observed between search interest and prescriptions for newer drugs, which indicates increasing usage, while older drugs with declining usage displayed limited correlation. Our findings suggest a growing role of non-physician professions in managing systemically progressed HCC within the US healthcare system, although oncologists remained primarily responsible for drug prescriptions.

**Conclusions:**

In conclusion, online search monitoring can offer the potential to reflect prescription trends specifically related to the treatment of HCC. This approach provides a swift and accessible means of evaluating the evolving landscape of HCC treatment.

## Introduction

Hepatocellular Carcinoma (HCC) is one of the leading causes of cancer-related deaths worldwide (1). Notably, most HCC patients are diagnosed in advanced disease stages, which contributes to the relatively high mortality rates (2, 3). Although hepatic resection is a potentially curative option for patients with limited-stage disease, patients with a large tumor burden who do not qualify for resection or who experience progression post-resection or after first-line treatment have dismal outcomes (4). To this end, systemic treatment approaches constitute an appropriate option for advanced and unresectable HCC (5–7). Over the past decades, there have been significant changes in guideline recommendations due to the emergence of immunotherapies and other targeted therapies. These changes have been driven by the introduction of sorafenib in 2006 (8), as well as positive results for several other targeted therapies and drug combinations (9, 10). As such, cytotoxic chemotherapy is no longer recommended because of its limited efficacy and undesirable side effects. Instead, immunotherapy-based combinations like atezolizumab plus bevacizumab, and tremelimumab plus durvalumab, have demonstrated favorable outcomes at a tolerable toxicity profile. Both combinations have shown promising results, leading to FDA approvals in 2020 and 2022 (11, 12). Despite the evolving landscape of HCC treatments and the improvements in guidelines recommendations, it however remains unclear to which extent these trends have been disseminated to the general public and how they have influenced prescription behaviors (4). This is particularly important given the increasing global incidence of HCC and the escalating influence of non-alcoholic steatohepatitis (NASH) as the fastest-growing underlying cause of HCC in the United States (13, 14).

Analyses of online search behavior have demonstrated the potential to mirror public health-related trends. For example, several studies have shown that online search behavior can forecast and track epidemiological parameters related to depression and cardiovascular disease (15, 16). Additionally, online search trends have been shown to be indicative of drug prescription trends across multiple drug classes, affirming the utility of this approach in mirroring drug usage patterns (17). Google Trends data, derived from the world’s leading search engine, can serve as a valuable resource for assessing the efficacy of public health interventions, primarily due to its broad accessibility (18). As such, Google search data can complement conventional epidemiological approaches and contribute to the monitoring of public health trends.

Given the advances in systemic treatment options for HCC and recent revisions in guideline recommendations, our study aims to offer a comprehensive overview of the fluctuations in online search activity and prescription patterns of drugs used for HCC treatment across various medical specialties. Additionally, we aimed to evaluate whether online search behavior can reflect changes in the prescription activity of HCC-directed drugs, thus reflecting adoption of drugs following authority approval.

## Methods

We queried drug prescriptions from the IQVIA National Prescription Audit (NPA). This database provides information on US outpatient prescription activity. NPA covers approximately 93% of nationwide outpatient prescription activity and uses that sample to project a reliable national estimate for all prescriptions dispensed in the US. Additional details of the data collection process can be found elsewhere (19). Prescription information were queried for drugs/drug combinations used as first line therapy for treatment of HCC (sorafenib, lenvatinib, bevacizumab/atezolizumab, tremelizumab/durvalumab); second line (cabozantinib, ramucirumab, regorafenib); or as further drugs for HCC treatment (pembrolizumab, nivolumab). Prescription activity was retrieved as total prescriptions of drugs across clinician specialties per month. Data on newly prescribed drugs (NRx), which include both new prescriptions as well as the renewal of expired prescriptions, were utilized to reflect changes in prescription patterns. For further analysis of drug combinations, individual data for each drug within the respective drug combination were utilized (instead of averaging data across multiple drug combinations). With respect to prescriber information, NPA-reported specialties nurse practitioners and physician assistants were grouped as Advanced Practice Providers (APP), and oncology and hematology specialists as oncology. We further categorized family practice, general practice, general preventive medicine, geriatrics, hepatology, internal medicine, internal medicine/pediatrics, osteopathic medicine, and pediatrics as internists.

Online search data were queried from Google Trends as query fractions per 10 million searches for drugs representative of HCC drug classes from January 1, 2017 to December 31, 2022 as previously described (17, 20). We queried selected terms representative for first-line therapy, second-line therapy, and other drugs used for HCC treatment. The queried drugs were identical to those extracted from the IQVIA database. We additionally extracted search data for both the generic drug name and the respective brand name for all relevant drugs. Queried brand names were: Cabometyx (cabozantinib), Lenvima (lenvatinib), Stivarga (regorafenib), Keytruda (pembrolizumab), Opdivo (nivolumab), Avastin (bevacizumab), Tecentriq (atezolizumab), Nexavar (sorafenib), Yervoy (ipilimumab), Cyramza (ramucirumab), Imjudo (tremelimumab), and Imfinzi (durvalumab). For an individual drug, analyses considered both brand names and generics as aggregate search data to provide a general estimate for public interest in the respective drug.

All analyses, including the calculation of Spearman’s rank correlation coefficients, were performed using Python 3.9.13 (Python Software Foundation, Delaware, USA) with Anaconda Distribution (version 2022.10) as well as the NumPy, Matplotlib and Pandas libraries.

## Results

Online search data were retrieved as queries per 10 million searches and prescriptions as the total number of newly dispensed drugs in the US. Data are subsequently shown according to the recommended treatment lines for HCC (6, 7). Overall, during our study period, monthly prescriptions of drugs used for HCC treatment increased by 173% between the first quarter (Q1) 2017 and Q4 2022 (1253 to 3422). In parallel, online searches increased by 3.5% (173–179 per 10 million).

### Drugs used in first-line treatment

**Figure 1:**
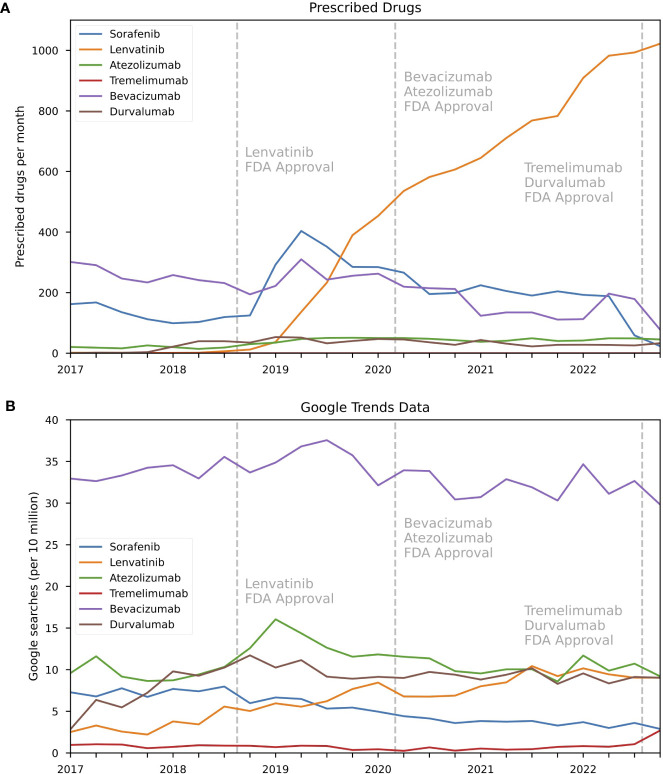
Trends in prescription and online search activity for first-line HCC drugs between 2017 and 2022 for the United States. **(A)** Prescription rates as quarterly average of prescription for HCC first-line drugs sorafenib, lenvatinib, atezolizumab, tremilimumab, bevacizumab, durvalumab and **(B)** corresponding online search volumes as searches per 10 million searches for aggregated brand and generic names.

Among first-line drugs, lenvatinib initially displayed minimal prescription activity, with six monthly prescriptions in Q3 2018 (**Figure 1A** and **Table 1**). However, there was a notable increase in prescription activity over time, with a 16,933% increase in prescriptions by Q4 2022 and reaching a monthly total of 1,022, eventually becoming the most used medication in this category. Simultaneously, there was a notable surge in online searches, exhibiting a growth rate of 62.3%, increasing from 5.57 to 9.04 searches per 10 million during the observed period (**Figure 1B**). In contrast, monthly prescriptions for sorafenib peaked in Q2 2019 with 404 monthly prescriptions, followed by a substantial decrease of 94.3% and ultimately reaching 23 monthly prescriptions in Q4 2022 (**Figure 1A**). Correspondingly, search volumes for sorafenib also declined by 55.5% (from 6.49 to 2.89 per 10 million searches), indicating reduced interest or awareness for this drug over time (**Figure 1B**). Additionally, prescription trends for atezolizumab and bevacizumab are depicted in **Figure 1A**. As for atezolizumab, there was a 10% decrease in monthly prescriptions between Q2 2020 and Q4 2022 (50 to 45 monthly prescriptions) after FDA approval for the combined use of bevacizumab and atezolizumab for HCC treatment (**Figure 1A**). This was paralleled by a decrease of 20.35% in search volumes (from 11.55 to 9.20 per 10 million searches) (**Figure 1B**). Tremelimumab and durvalumab received approval for HCC treatment in late 2022, and did not qualify for correlation analysis. However, tremelimumab exhibited an increased search activity of 155% between Q4 2022 and Q3 2022 (2.70 vs. 1.06 per 10 million searches). Correlations between prescription trends and online search activity from 2017 to 2022 are shown in **Table 2**. Notably, the strongest correlation among the first-line drugs was observed for lenvatinib (r=0.90), followed by a less strong correlation for bevacizumab (r=0.45) and atezolizumab (r=0.33). Conversely, for sorafenib, which represented a drug with an overall decreasing prescription activity, no correlation was observed (r= -0.13).

**Table 1:** Annual trends for prescriptions of drugs used for HCC treatment.

Year	Bevaci-zumab	Atezo-lizumab	Lenva-tinib	Sorafenib	Cabozan-tinib	Ramu-cirumab	Regora-fenib	Pembro-lizumab	Durva-lumab	Nivo-lumab
2017	3217	242	18	1732	6627	187	1143	641	19	1527
2018	2776 (-13.7%)	249 (+2.9%)	64 (+255.6%)	1338 (-22.7%)	10816 (+63.2%)	200 (+7.0 %)	936 (-18.1%)	1174 (+83.2%)	408 (+2047.4%)	1822 (+19.3%)
2019	3093 (+11.4%)	550 (+120.8%)	2388 (+3631.3%)	3999 (+198.9%)	11988 (+10.8%)	250 (+25%)	3736 (+299.1%)	1738 (+48.0%)	534 (+30.9%)	1703 (-6.5%)
2020	2727 (-11.8%)	572 (+4.0%)	6532 (+173.5%)	2834 (-29.1%)	10335 (-13.8%)	151 (-39.6%)	4450 (+19.1%)	2264 (+30.3%)	468 (-12.4%)	1317 (-22.7%)
2021	1512 (-44.6%)	505 (-11.7%)	8721 (+33.5%)	2473 (-12.7%)	14368 (+39.0%)	205 (+35.8%)	4622 (+3.9%)	2519 (+11.3%)	376 (-19.7%)	1163 (-11.7%)
2022	1698 (+12.3%)	557 (+10.3%)	11719 (+34.4%)	1390 (-43.8%)	17041 (+18.6%)	119 (-42.0%)	4786 (+3.5%)	2776 (+10.2%)	342 (-9.0%)	1311 (+12.7%)

**Table 2:** Correlation coefficients for prescriptions and online search volumes for drugs used for HCC treatment.

Category	Drugs	Correlation coefficient
First-line	Lenvatinib	0.90
	Bevacizumab	0.45
	Atezolizumab	0.33
	Sorafenib	-0.13
	Tremilimumab	*
	Durvalumab	*
Second-line	Cabozantinib	0.67
	Ramucirumab	0.23
	Regorafenib	-0.06
Other drugs	Pembrolizumab	0.54
	Nivolumab	0.36

*no correlation data available.

### Drugs used for second or later treatment lines

**Figure 2:**
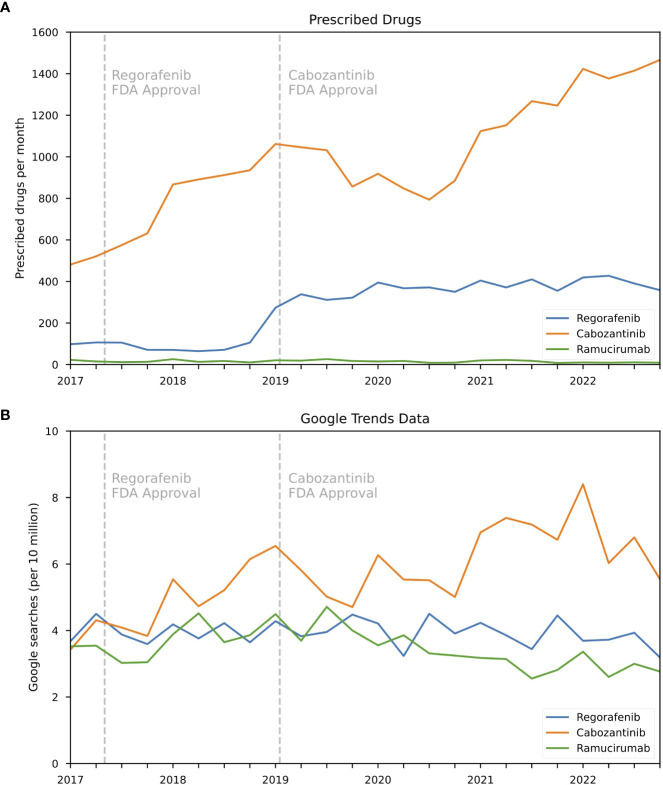
Trends in prescription and online search activity for second-line HCC drugs between 2017 and 2022 for the United States. **(A)** Prescription rates as quarterly average of prescription for HCC second-line drugs regorafenib, cabozantinib, ramucirumab and **(B)** corresponding online search volumes as searches per 10 million searches for aggregated brand and generic names.

Next, data for second-line prescription treatments for HCC were queried, including cabozantinib, regorafenib, and ramucirumab. Among these, cabozantinib stood out with the highest prescription rate and search volumes (**Figures 2A, B**). Monthly prescriptions for cabozantinib increased by 38% (from 1,062 in Q1 2019 to 1,466 in Q4 2022) (**Figure 2A**), which coincided with a 15.4% decrease in online search activity (**Figure 2B**). Search activity for cabozantinib reached its peak in Q1 2022 but has since experienced a decline. Prescriptions for ramucirumab decreased by 52.6% from 19 to 9 between Q2 2019 and Q4 2022 (**Figure 2A**), which was paralleled by a 24.9% decline in online search activity (3.69 to 2.77 per 10 million searches) (**Figure 2B**). Regorafenib prescriptions increased by 237.7% between Q2 2017 and Q4 2022 (106 to 358) (**Figure 2A**) with a corresponding decrease of 29.1% in search volume (4.50 to 3.19 per 10 million searches) (**Figure 2B**). The overall correlation coefficients were 0.67, 0.23, and -0.06 for cabozantinib, ramucirumab, and regorafenib, respectively (**Table 2**).

Additional drugs for second-line HCC treatment and beyond are shown in **Supplementary Figure 1**. As such, pembrolizumab prescriptions increased by 146.5% after FDA approval in Q4 2018 until Q4 2022 (101 to 249 monthly prescriptions) (**Supplementary Figure 1A**). Correspondingly, online searches increased by 13.75% (from 60.09 to 68.35 per 10 million searches) during the same period (**Supplementary Figure 1B**). In contrast, nivolumab prescriptions decreased by 4.3% from Q3 2017 to Q4 2022 (117 to 112 monthly prescriptions) (**Supplementary Figure 1A**) with a corresponding decrease of 41.47% in search volume (43.81 to 25.64 per 10 million searches) (**Supplementary Figure 1B**). Correlation coefficients between online searches and prescription activity for pembrolizumab were 0.54, while a correlation coefficient of 0.36 was noted for nivolumab (**Table 2**).

### Prescription activity across specialties

**Figure 3:**
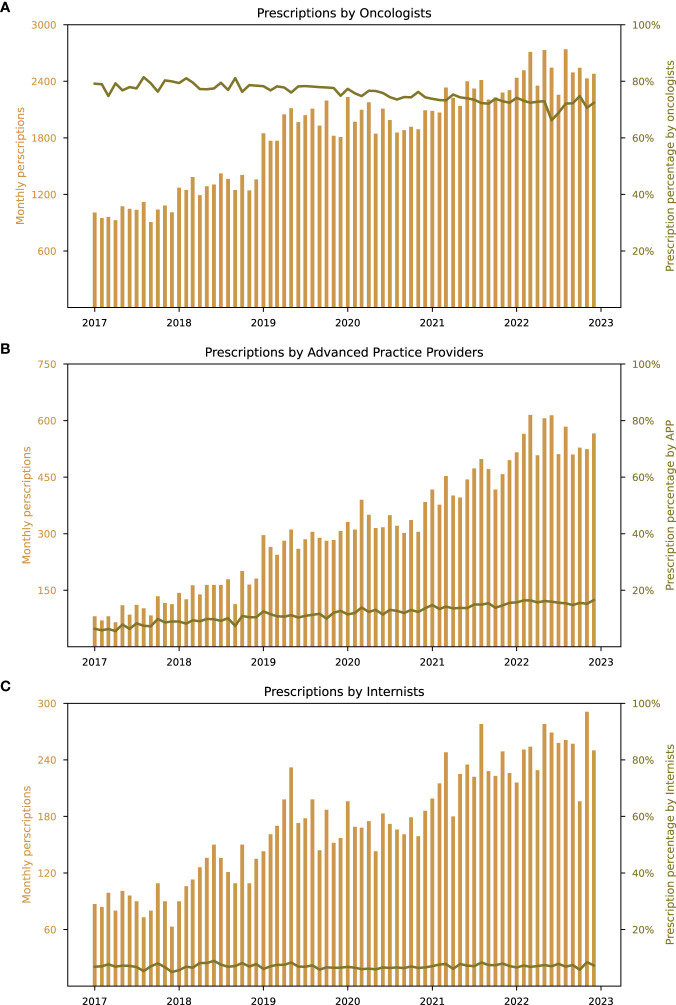
Prescription activity for drugs used for HCC treatment across specialties between 2017 and 2022. Absolute numbers for HCC drug prescriptions and relative proportion in % of all HCC drug for the following selected specialties **(A)** oncologists, **(B)** advanced practice providers and **(C)** internists.

**Figure 4:**
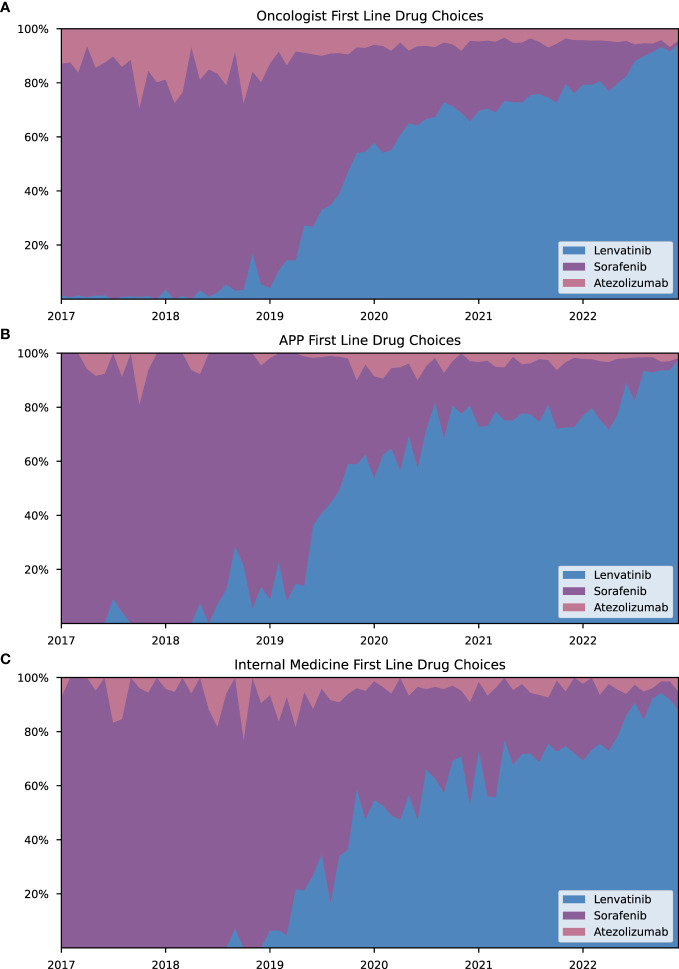
First-line drug choices for HCC treatment across specialties. Monthly share for approved first-line drugs (sorafenib, lenvatinib, atezolizumab) as a proportion of aggregated prescriptions of all first-line HCC drugs. Data are shown in % for the following selected specialties **(A)** oncologists, **(B)** advanced practice providers and **(C)** internists. Share of atezolizumab in prescriptions were considered representative for the combination therapy atezolizumab/bevacizumab.

**Figure 5:**
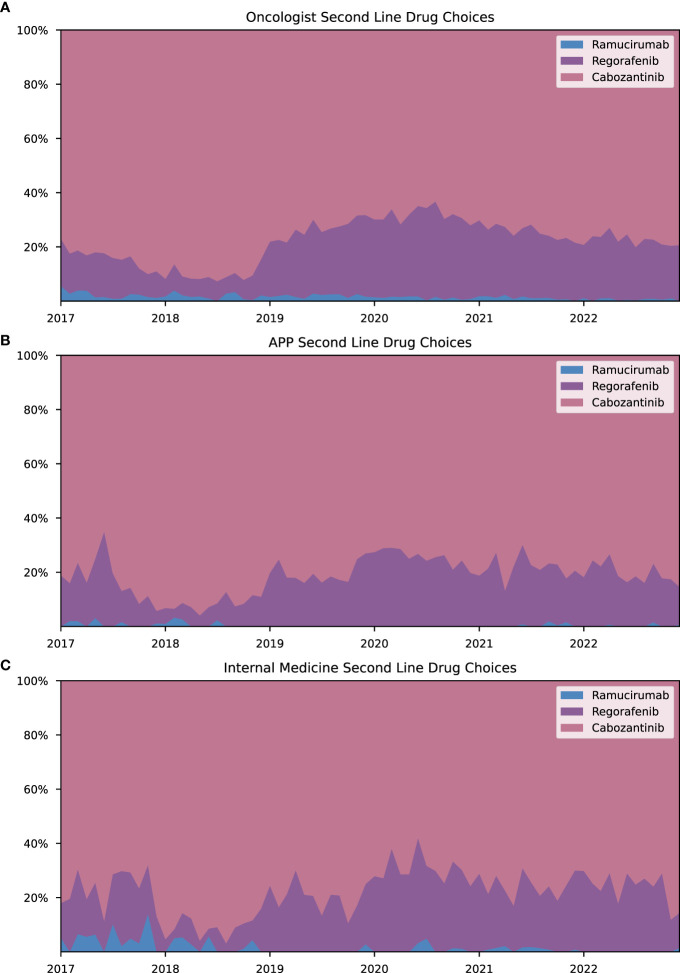
Second-line drug choices for HCC treatment across specialties. Monthly share for approved second-line drugs (regorafenib, cabozantinib, ramucirumab) as a proportion of aggregated prescriptions of all second-line HCC drugs. Data are shown in % for the following selected specialties **(A)** oncologists, **(B)** advanced practice providers and **(C)** internists.

Most prescriptions for HCC drugs were issued by oncologists, APPs, and internists. Oncologists accounted for the largest share of prescriptions between 2017 and 2022, although their proportion decreased from 79.17% in January 2017 to 72.40% in December 2022 (**Figure 3A**). In contrast, APPs increased their share from 6.37% in January 2017 to 16.53% in December 2022 (**Figure 3B**). The proportion of prescriptions by internists remained relatively constant, starting with 6.84% in January 2017 and increased to 7.30% in 2022 (**Figure 3C**). Drug choice trends for both first- and second-line treatments exhibited similar patterns across the three primary prescribing specialties, as shown in **Figures 4**, **5**. Notably, lenvatinib accounted for the largest share of prescriptions after its approval in 2018, while the shares of sorafenib and atezolizumab decreased significantly (**Figure 4**). Cabozantinib emerged as the most prescribed second-line drug across all specialties, followed by regorafenib. There were only few prescriptions for ramucirumab (**Figure 5**). As additionally shown in **Supplementary Figure 2**, APPs had a greater involvement in prescribing orally administered HCC drugs (lenvatinib, cabozantinib, sorafenib, regorafenib) compared to intravenous administered HCC drugs (atezolizumab, pembrolizumab, nivolumab, ramucirumab) (14.1% vs. 5.9%). These findings suggest that APPs may play a more prominent role in prescribing small molecule inhibitors.

## Discussion

Our study provides a comprehensive analysis of prescription rates and online search volumes, as an indicator of public awareness, for drugs used in the treatment of HCC between 2017 and 2022. Notably, clear correlations between prescription rates and online searches were observed for a select group of HCC drugs. The correlation between prescription trends and online search activity was most pronounced for drugs that had an overall increasing trend. This was particularly notable for lenvatinib and cabozantinib. In contrast, drugs with decreasing prescriptions rates or those with relatively low prescription activity demonstrated minimal to no correlation.

This study is the first to both analyze prescription drug usage for treatment of HCC and evaluate the potential of online search monitoring to understand therapeutic adoption of updated guidelines for systemic HCC therapy. Despite the approval of several drugs in newer years, including the FDA’s October 2022 approval of the combination tremelimumab with durvalumab, effective monitoring of prescription activity changes beyond official prescription accounts has been lacking (21). Our data demonstrate the potential of Google search analyses to offer near real-time monitoring and a potential to complement conventional prescription trends.

Since its approval in 2018, lenvatinib was broadly applied for HCC treatment. Its exceptionally high correlation of 0.90 suggests that Google trends analysis is well-suited to provide a qualitative assessment of the drug’s adoption. Atezolizumab’s correlation of 0.33 also suggests that the first-line combination of atezolizumab with bevacizumab might also be represented by our Google search analysis. However, with respect to lenvatinib, overall trends were similar but less strong. Sorafenib did not show any correlation, suggesting that drugs of declining usage did not show signs of correlation and are therefore more likely to stabilize or show a slight decrease in online searches. For example, a previous study dealing with prescription trends of SGLT2 inhibitors and GLP1 receptor agonists reported similar association patterns (17). Thus, the increase in tremelimumab queries, as noted in Q4 2022, may precede a potential future rise in prescriptions.

Cabozantinib, the most applied drug in second-line treatment as well as pembrolizumab, frequently applied beyond first- or second-line treatment, showed highest correlation. However, in absolute terms the degree of correlations was relatively lower when compared to lenvatinib.

Treatment guidelines play an important role in informing prescribing decisions by providing recommendations for the use of specific drugs in the management of particular medical conditions. Lenvatinib and cabozantinib have rapidly integrated into guidelines for renal cell carcinoma (RCC) and HCC (22, 23). Notably, NCCN guidelines introduced cabozantinib for advanced HCC in 2019 (22) and lenvatinib for unresectable HCC in 2018 (24). Consistent with these updates, our data show parallel surges in prescriptions and online searches post-approval and guideline revisions.

Overall, these findings further underscore the observation that online search activity might predict the future prescription distribution of drugs applied for HCC treatment. In terms of prescribing specialties, APP experienced the most prominent increase with 2.6-fold higher prescription activity for APP-related specialties during the study period. This finding particularly highlights the growing involvement of non-physician professions in HCC treatment within the US healthcare system. As such, APP like nurse practitioners and physician assistants are increasingly vital members of oncology teams in the United States (25, 26). Our data indicated that there were no relevant differences in HCC prescription activities between medical specialties and APP. Thus, the present analysis highlights the potential efficacy of HCC treatment delivery by APPs. This underscores the well-established benefits of enhanced access and cost-effectiveness associated with APP involvement in the management of oncology patients (27). This study has limitations. Prescription data provided by the National Prescription Audit is sampled from outpatient pharmacies only and extrapolated to estimate all prescriptions, which may allow for some errors. The IQVIA NPA database may not be comprehensive, as it excludes prescriptions filled at hospitals, clinics, or mail-order pharmacies. Data are provided as continuously updated numbers on a monthly basis, potentially lagging behind the latest prescription trends. Thus, this study does not capture the complete picture of all prescriptions, but rather focuses on a subset of new prescriptions. Nevertheless, this approach is particularly sensitive to changes in guidelines and drug approvals. Drugs analyzed in this study are not limited in their use cases to therapy of HCC. For some drugs, most of their prescriptions could be attributed to other disease types. Lastly, there is no information regarding the users utilizing drug internet searches. It is not clear whether search requests are generated by professionals, patients, or another unknown group. Demographic characteristics of those individuals most prone to utilizing Google Technology, such as IT capability and age, may influence results and distort this study’s findings. The absence of demographic data on online search activities overall generalizability might constitute a potential limitation. For future research, incorporating user surveys or platform partnerships might allow for more nuanced analyses across diverse populations. Additionally, spikes in search queries could be driven by factors other than prescription increase, such as media coverage or social media trends. In terms of correlation analyses, correlative trends between Google Trends data and prescription trends do not imply causation.

This study comprehensively provides trends in prescriptions and online search activity for drugs used for HCC treatment. Our study reveals striking correlations between online search trends and prescribed HCC treatments, suggesting that online search data can be a powerful tool for tracking evolving treatment landscapes, especially for newly approved drugs or those with changing guidelines. Thus, our data underscore the value of utilizing online search data as a highly effective and timely tool for gaining insights into the evolving landscape of HCC treatments.

## Data availability statement

The original contributions presented in the study are included in the article/**Supplementary Material**. Further inquiries can be directed to the corresponding author.

## Author contributions

PB: Writing – original draft. AS: Writing – original draft. RA: Writing – review & editing. AR: Writing – review & editing. FC: Writing – review & editing. JE: Writing – review & editing. SS: Writing – review & editing. DS: Writing – review & editing. HV: Writing – review & editing. HS: Writing – review & editing. JF: Writing – review & editing. OD: Writing – original draft.

## References

[1] Sung H Ferlay J Siegel RL Laversanne M Soerjomataram I Jemal A. Global cancer statistics 2020: GLOBOCAN estimates of incidence and mortality worldwide for 36 cancers in 185 countries. CA Cancer J Clin (2021) 71(3):209–49. doi: 10.3322/caac.21660 33538338

[2] Altekruse SF McGlynn KA Reichman ME. Hepatocellular carcinoma incidence, mortality, and survival trends in the United States from 1975 to 2005. J Clin Oncol (2009) 27(9):1485–91. doi: 10.1200/JCO.2008.20.7753 PMC2668555 19224838

[3] Park JW Chen M Colombo M Roberts LR Schwartz M Chen P-J. Global patterns of hepatocellular carcinoma management from diagnosis to death: the BRIDGE Study. Liver Int (2015) 35(9):2155–66. doi: 10.1111/liv.12818 PMC4691343 25752327

[4] Reig M Forner A Rimola J Ferrer-Fàbrega J Burrel M Garcia-Criado Á. BCLC strategy for prognosis prediction and treatment recommendation: The 2022 update. J Hepatol (2022) 76(3):681–93. doi: 10.1016/j.jhep.2021.11.018 PMC8866082 34801630

[5] Bruix J Chan SL Galle PR Rimassa L Sangro B. Systemic treatment of hepatocellular carcinoma: An EASL position paper. J Hepatol (2021) 75(4):960–74. doi: 10.1016/j.jhep.2021.07.004 34256065

[6] Vogel A Martinelli E. Updated treatment recommendations for hepatocellular carcinoma (HCC) from the ESMO Clinical Practice Guidelines. Ann Oncol (2021) 32(6):801–5. doi: 10.1016/j.annonc.2021.02.014 33716105

[7] Benson AB D’Angelica MI Abbott DE Anaya DA Anders R Are C. Hepatobiliary cancers, version 2.2021, NCCN clinical practice guidelines in oncology. J Natl Compr Canc Netw (2021) 19(5):541–65. doi: 10.6004/jnccn.2021.0022 34030131

[8] Wilhelm S Carter C Lynch M Lowinger T Dumas J Smith RA. Discovery and development of sorafenib: a multikinase inhibitor for treating cancer. Nat Rev Drug Discovery (2006) 5(10):835–44. doi: 10.1038/nrd2130 17016424

[9] Kudo M Finn RS Qin S Han K-H Ikeda K Piscaglia F. Lenvatinib versus sorafenib in first-line treatment of patients with unresectable hepatocellular carcinoma: a randomised phase 3 non-inferiority trial. Lancet (2018) 391(10126):1163–73. doi: 10.1016/S0140-6736(18)30207-1 29433850

[10] Finn RS Qin S Ikeda M Galle PR Ducreux M Kim T-Y. Atezolizumab plus bevacizumab in unresectable hepatocellular carcinoma. N Engl J Med (2020) 382(20):1894–905. doi: 10.1056/NEJMoa1915745 32402160

[11] Salem R Li D Sommer N Hernandez S Verret W Ding B. Characterization of response to atezolizumab + bevacizumab versus sorafenib for hepatocellular carcinoma: Results from the IMbrave150 trial. Cancer Med (2021) 10(16):5437–47. doi: 10.1002/cam4.4090 PMC8366100 34189869

[12] Kelley RK Sangro B Harris W Ikeda M Okusaka T Kang Y-K. Safety, efficacy, and pharmacodynamics of tremelimumab plus durvalumab for patients with unresectable hepatocellular carcinoma: randomized expansion of a phase I/II study. J Clin Oncol (2021) 39(27):2991–3001. doi: 10.1200/JCO.20.03555 34292792 PMC8445563

[13] Estes C Razavi H Loomba R Younossi Z Sanyal AJ. Modeling the epidemic of nonalcoholic fatty liver disease demonstrates an exponential increase in burden of disease. Hepatology (2018) 67(1):123–33. doi: 10.1002/hep.29466 PMC5767767 28802062

[14] Llovet JM Kelley RK Villanueva A Singal AG Pikarsky E Roayaie S. Hepatocellular carcinoma. Nat Rev Dis Primers (2021) 7(1):6. doi: 10.1038/s41572-020-00240-3 33479224 PMC13537433

[15] Wang A McCarron R Azzam D Stehli A Xiong G DeMartini J. Utilizing big data from google trends to map population depression in the United States: exploratory infodemiology study. JMIR Ment Health (2022) 9(3):e35253. doi: 10.2196/35253 35357320 PMC9015761

[16] Senecal C Mahowald M Lerman L Lopes-Jimenez F Lerman A. Increasing utility of Google Trends in monitoring cardiovascular disease. Digit Health (2021) 7:20552076211033420. doi: 10.1177/20552076211033420 34873449 PMC8642777

[17] Dzaye O Berning P Razavi AC Adhikari R Jha K Nasir K. Online searches for SGLT-2 inhibitors and GLP-1 receptor agonists correlate with prescription rates in the United States: An infodemiological study. Front Cardiovasc Med (2022) 9:936651. doi: 10.3389/fcvm.2022.936651 35966558 PMC9372305

[18] Arora VS McKee M Stuckler D. Google Trends: Opportunities and limitations in health and health policy research. Health Policy (2019) 123(3):338–41. doi: 10.1016/j.healthpol.2019.01.001 30660346

[19] Turner LW Nartey D Stafford RS Singh S Alexander GC. Ambulatory treatment of type 2 diabetes in the U.S., 1997-2012. Diabetes Care (2014) 37(4):985–92. doi: 10.2337/dc13-2097 PMC4178325 24198301

[20] Berning P Huang L Razavi AC Boakye E Osuji N Stokes AC. Association of online search trends with vaccination in the United States: June 2020 through May 2021. Front Immunol (2022) 13:884211. doi: 10.3389/fimmu.2022.884211 35514956 PMC9066639

[21] Keam SJ. Tremelimumab: first approval. Drugs (2023) 83(1):93–102. doi: 10.1007/s40265-022-01827-8 36571670

[22] Benson AB D’Angelica MI Abrams T Abbott DE Ahmed A Anaya DA. NCCN guidelines^®^ Insights: biliary tract cancers, version 2.2023: featured updates to the NCCN guidelines. J Natl Compr Cancer Network (2023) 21(7):694–704. doi: 10.6004/jnccn.2023.0035 37433432

[23] Motzer RJ Jonasch E Agarwal N Alva A Baine M Beckermann K. Kidney cancer, version 3.2022, NCCN clinical practice guidelines in oncology. J Natl Compr Cancer Network (2022) 20(1):71–90. doi: 10.6004/jnccn.2022.0001 PMC10191161 34991070

[24] Benson AB D’Angelica MI Abbott DE Abrams TA Alberts SR Anaya DA. NCCN guidelines insights: Hepatobiliary cancers, version 2.2019: Featured updates to the NCCN guidelines. J Natl Compr Cancer Network (2019) 17(4):302–10. doi: 10.6004/jnccn.2019.0019 30959462

[25] Bruinooge SS Pickard TA Vogel W Hanley A Schenkel C Garrett-Mayer E. Understanding the role of advanced practice providers in oncology in the United States. J Oncol Pract (2018) 14(9):e518–32. doi: 10.1200/JOP.18.00181 30133346

[26] Pickard T Williams S Tetzlaff E Petraitis C Hylton H. Team-based care in oncology: the impact of the advanced practice provider. Am Soc Clin Oncol Educ Book (2023) 43:e390572. doi: 10.1200/EDBK_390572 37279437

[27] Cairo J Muzi MA Ficke D Ford-Pierce S Goetzke K Stumvoll D. Practice model for advanced practice providers in oncology. Am Soc Clin Oncol Educ Book (2017) 37:40–3. doi: 10.1200/EDBK_175577 28561709

