# Supplementary material for: Online searches for hepatocellular carcinoma drugs mirror prescription trends across specialties and changes in guideline recommendations
Source: Front Oncol. 2024 Feb 9;14:1324095. doi: 10.3389/fonc.2024.1324095 (PMC10884243; doi:10.3389/fonc.2024.1324095)

**Supplementary Material**

**Online Searches for Hepatocellular Carcinoma Drugs Mirror Prescription Trends Across Specialties and Changes in Guideline Recommendations**

Philipp Berning MD^1^, Adrian E. Schroer^1^, Rishav Adhikari MD^1^, Alexander C. Razavi^1^, Francois H. Cornelis MD PhD^2^, Joseph P. Erinjeri MD PhD^2^, Stephen B. Solomon MD^2^, Debkumar Sarkar DO^2^, Hebert Alberto Vargas MD^3^, Heiko Schöder MD^2^, Josef J. Fox MD^2^, Omar Dzaye MD MPH PhD^1,2^

^1^ Johns Hopkins Ciccarone Center for the Prevention of Cardiovascular Disease, Johns Hopkins University School of Medicine, Baltimore, MD, USA

^2^ Department of Radiology, Memorial Sloan Kettering Cancer Center, New York, NY, USA

^3^ Department of Radiology, NYU Langone, New York, NY, USA

**Supplementary Figure 1. Trends in prescription and online search activity for additional drugs in HCC treatment between 2017 and 2022 for the United States.**


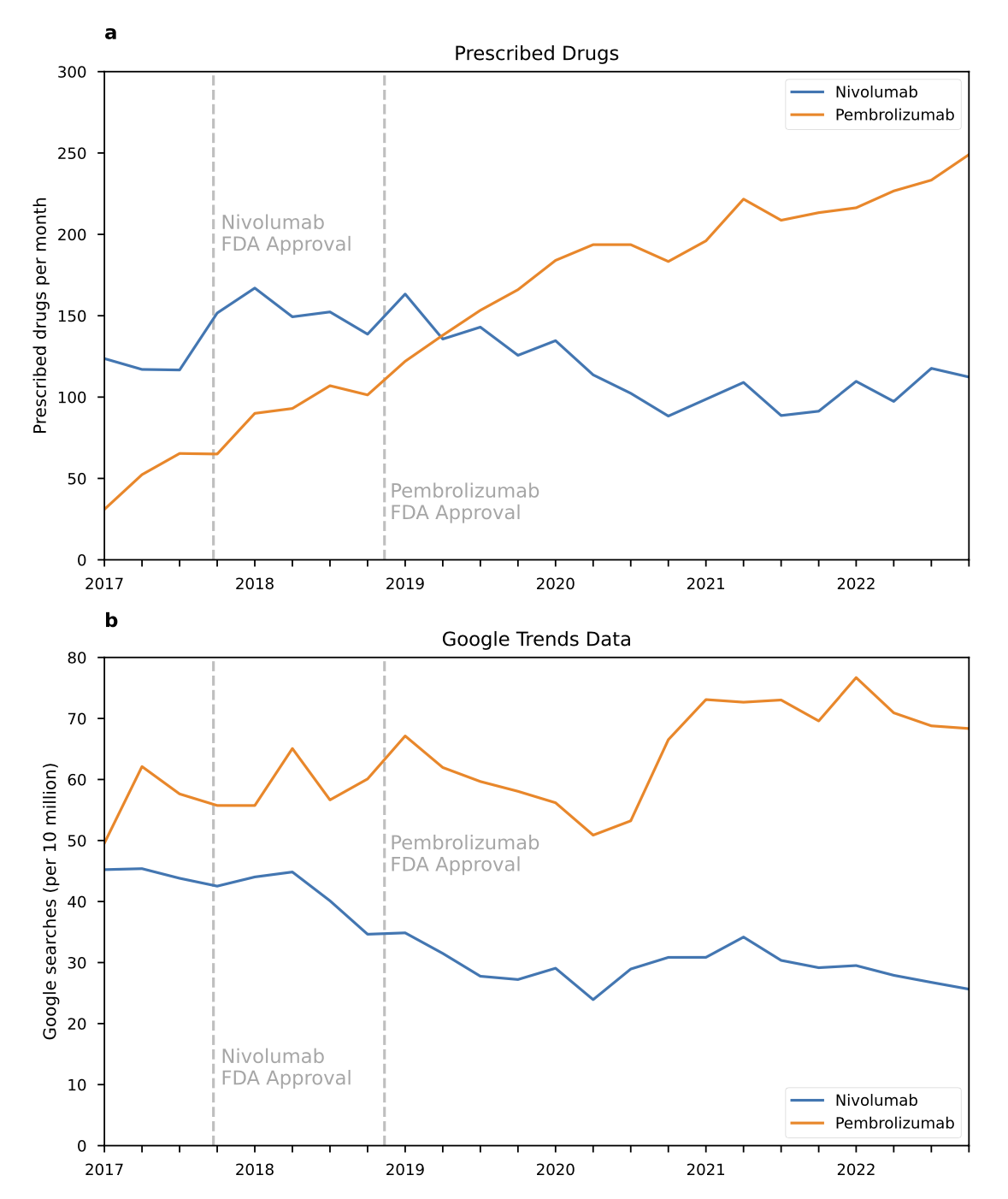


**Supplementary Figure 2. Prescription distributions of prescriptions for orally and intravenously applied HCC drugs among oncologists, APP and other specialties.**


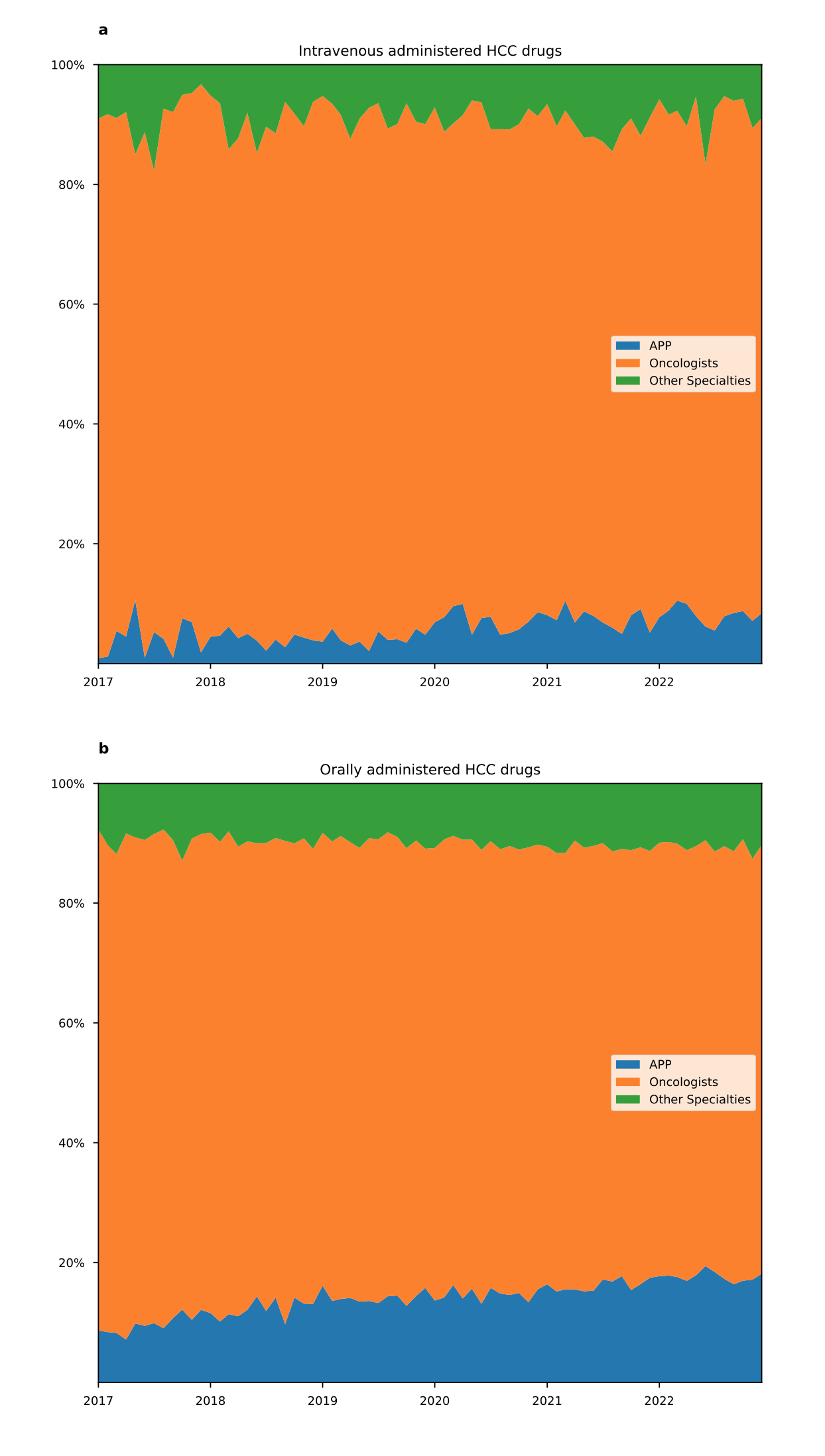

Supplement: Supplementary file 1 [file DataSheet_1.docx]
